# *rpoB* Mutations Causing Discordant Rifampicin Susceptibility in *Mycobacterium tuberculosis*: Retrospective Analysis of Prevalence, Phenotypic, Genotypic, and Treatment Outcomes

**DOI:** 10.1093/ofid/ofz065

**Published:** 2019-02-12

**Authors:** Nomonde R Mvelase, Melendhran Pillay, Wilbert Sibanda, Jacqueline N Ngozo, James C M Brust, Koleka P Mlisana

**Affiliations:** 1 Department of Medical Microbiology, KwaZulu-Natal Academic Complex, National Health Laboratory Service, Durban, South Africa; 2 Department of Medical Microbiology, School of Laboratory Medicine and Medical Sciences, University of KwaZulu-Natal, Durban, South Africa; 3 Department of Biostatistics, School of Nursing and Public Health, University of KwaZulu-Natal, Durban, South Africa; 4 Centre for the AIDS Programme of Research in South Africa (CAPRISA), University of KwaZulu-Natal, Durban, South Africa; 5 Department of Health, KwaZulu-Natal Province, Pietermaritzburg, South Africa; 6 Department of Medicine, Albert Einstein College of Medicine and Montefiore Medical Center, Bronx, New York

**Keywords:** discordant, rifampicin, *rpoB* mutations, TB

## Abstract

**Background:**

Discordant genotypic/phenotypic rifampicin susceptibility testing in *Mycobacterium tuberculosis* is a significant challenge, yet there are limited data on its prevalence and how best to manage such patients. Whether to treat isolates with *rpoB* mutations not conferring phenotypic resistance as susceptible or multidrug-resistant tuberculosis (MDR-TB) is unknown. We describe phenotypic and genotypic characteristics of discordant isolates and clinical characteristics and treatment outcomes of affected patients in KwaZulu-Natal, South Africa.

**Methods:**

We analyzed clinical isolates showing rifampicin resistance on GenoType MTBDR*plus* while susceptible on 1% agar proportion method. We measured rifampicin minimum inhibitory concentrations (MICs) using Middlebrook 7H10 agar dilution and BACTEC MGIT 960. Sensititre MYCOTB plates were used for drug-susceptibility testing, and *rpoB* gene sequencing was performed on all isolates. Local MDR-TB program data were reviewed for clinical information and patient outcomes.

**Results:**

Discordant isolates constituted 4.6% (60) of 1302 rifampicin-resistant cases over the study period. Of these, 62% remained susceptible to isoniazid and 98% remained susceptible to rifabutin. Rifampicin MICs were close to the critical concentration of 1 µg/mL (0.5–2 µg/mL) for 83% of isolates. The most frequent *rpoB* mutations were Q513P (25.3%), D516V (19.2%), and D516Y (13.3%). Whereas 70% were human immunodeficiency virus infected, the mean CD4 count was 289 cells/mm^3^ and 87% were receiving antiretroviral therapy. Standard therapy for MDR-TB was used and 53% achieved successful treatment outcomes.

**Conclusions:**

Rifampicin-discordant TB is not uncommon and sequencing is required to confirm results. The high susceptibility to rifabutin and isoniazid and poor treatment outcomes with the current regimen suggest a potential utility for rifabutin-based therapy.

Tuberculosis (TB) drug-susceptibility testing (DST) has become increasingly important due to the escalating resistance to TB drugs. Conventional phenotypic DST is considered the gold standard for DST, but it remains very slow, taking weeks to months to give final results. As a result, the World Health Organization (WHO) has endorsed the use of molecular tests MTBDR*plus* (GenoType MTBDR*plus* assay; Hain Lifescience, Nehren, Germany), Xpert MTB/RIF (Cepheid), and Nipro NTM+MDRTB detection kit 2 (Tokyo, Japan) for the initial diagnosis of drug-resistant TB [1, 2]. These tests detect rifampicin resistance by identifying resistance-conferring mutations in the 81-base pair region of the *rpoB* gene (also called rifampicin resistance determining region [RRDR]). Mutations in the RRDR are responsible for more than 95% of resistance to rifampicin; therefore, molecular tests have proven to be highly sensitive in the diagnosis of rifampicin resistance [3–5]. Although initially considered to be highly specific, the emergence of RRDR mutations that do not demonstrate phenotypic resistance (herein referred to as discordant) have cast doubt on the reliability of molecular tests in detecting rifampicin resistance [6–8]. This phenomenon occurs more frequently with liquid-based than with solid-based DST [6, 7].

In South Africa, both the Xpert MTB/RIF (Xpert) and MTBDR*plus* are available for the rapid diagnosis of drug-resistant TB, but phenotypic DST is still used in our laboratory to confirm rifampicin resistance and to establish susceptibility to additional anti-TB drugs. Discordant results between molecular and phenotypic rifampicin susceptibility testing are often encountered, causing diagnostic and clinical management dilemmas. In a study conducted in Haiti, a region with high TB and human immunodeficiency virus (HIV) prevalence like South Africa, discordant rifampicin mutations constituted 10% of all rifampicin-resistant cases [9]. Observational studies have demonstrated that these mutations may be clinically significant, especially when associated with resistance to other anti-TB drugs [10–12].

The province of KwaZulu-Natal, South Africa has one of the largest burdens of TB in the world, with an incidence rate of 781 cases/100 000 population in 2016. To date, however, no study has examined the prevalence, the genotypic and phenotypic characteristics, or the treatment outcomes of rifampicin discordant TB in this region. Understanding these characteristics is important to ensure optimal diagnosis and treatment to ensure better treatment outcomes and to prevent ongoing transmission.

## METHODS

### Study Design and Setting

This is a retrospective analysis of stored clinical isolates and TB data collected for routine programmatic management. The study was conducted at the central academic laboratory, which services the public sector of KwaZulu-Natal (KZN) province. Although the KZN province is the second most populous in the country with just over 11 million people, it carries one third of the country’s drug-resistant TB burden with 6630 and 754 documented cases of MDR and extensively drug-resistant (XDR) TB in 2012, respectively [13].

The study was conducted after Xpert testing had been rolled out in the country. Tuberculosis culture was therefore limited to those patients suspected of having paucibacillary TB that may have been missed by Xpert, rifampicin-resistant TB detected using Xpert, and treatment failures. Cultures were performed using automated BACTEC Mycobacteria Growth Indicator Tube (MGIT) 960 system (Becton Dickinson). Indirect line probe assay (LPA) using the MTBDR*plus* version 2 assay was performed on all positive MGIT cultures using standard methods. Additional DST (isoniazid [INH], rifampicin, ofloxacin, streptomycin, and kanamycin) using 1% agar proportion method (APM) on Middlebrook 7H10 was done for all cases with resistance to rifampicin or INH on the MTBDR*plus*.

### Isolates

Consecutive *Mycobacterium tuberculosis* clinical isolates showing rifampicin resistance on the MTBDR*plus* 2.0 while susceptible on the APM using Middlebrook 7H10 agar were used for this study. The MTBDR*plus* test is based on deoxyribonucleic acid (DNA) strip technology with membrane strips that are coated with specific probes complementary to the amplified nucleic acids. The strip contains both wild-type and mutation probes. Resistance is detected by binding to one of the mutation probes or by absence of binding to one or more wild-type probes. Mutation probes detect only the most common resistance-conferring mutations (S531L, H526Y, H526D, and D516V). The prevalence of discordant TB was determined by calculating the rate of discordant isolates among all rifampicin-resistant TB detected using the MTBDR*plus* 2.0 between May and December 2014. In addition to these isolates, we included a group of randomly selected stored discordant isolates that had been routinely collected between 2013 and 2014. We also included a time-matched group of 40 wild-type isolates for comparison.

#### Agar Dilution Minimum Inhibitory Concentration

Rifampicin powder was purchased from Sigma and dissolved in dimethyl sulfoxide. The solution was serially diluted 2-fold with distilled water before it was added into the 7H10 agars so as to achieve rifampicin concentrations of 4, 2, 1, 0.5, 0.25, and 0.125 µg/mL. One quadrant in each 7H10 plate was used as a control by pouring 7H10 agar medium without the drug. The prepared bacterial inoculum was diluted 1:100 dilution, and a 100 µL of this suspension was inoculated into each 7H10 plate containing different rifampicin concentrations plus the control quadrant. The plates were incubated for 3 weeks at 37ºC in an atmosphere of 5% to 10% CO_2_. Each isolate was tested in triplicate, and the H37Rv control (ATCC 25177) was included with every minimum inhibitory concentration (MIC) experiment, and the results were acceptable if the H37Rv strain showed susceptible results (critical concentration of rifampin 1 μg/mL) [14]. The MIC was defined as the lowest concentration where there was less than 1% of growth on the antibiotic-containing quadrant when compared with the control quadrant. This test was performed on all discordant isolates including the 40 fully susceptible isolates.

#### Mycobacteria Growth Indicator Tube Minimum Inhibitory Concentration

The BACTEC MGIT 960 system was used to obtain the MIC. Rifampicin stock solutions were prepared to achieve 2-fold serial dilutions of 0.125, 0.25, 0.5, 1, and 2 µg/mL. A drug volume of 100 µL was added into the MGIT tube supplemented with 0.8 mL OADC. The prepared inoculum was diluted 1:5 and 500 µL was added into the MGIT tube. Half a milliliter of the 1:100 dilution of the inoculum was inoculated into a drug-free growth control tube. Each isolate was tested in duplicate and the H37Rv control was included with every MIC experiment. All tubes were loaded into the MGIT instrument and read at 2 weeks. The MIC was defined as the lowest concentration that was negative when the growth control turned positive. The results were considered acceptable if the H37Rv strain was susceptible to rifampicin using a critical concentration of 1 μg/mL rifampin [14].

#### Sensititre MYCOTB Minimum Inhibitory Concentration

The Sensititre MYCOTB plate (TREK Diagnostics) is a commercially available, 96-well microdilution plate containing 12 lyophilized anti-TB drugs over a range of 7 to 8 concentrations. The MIC was determined following the manufacturer’s instructions. An H37Rv control was set up with each batch of testing, and the results were interpreted by at least 2 independent readers. The MIC was defined as the lowest drug concentration with no visible growth. The following critical concentrations were used to interpret the results: 1.0 for rifampicin, 0.5 for rifabutin, 0.25 for INH, 5.0 for ethambutol, 1.0 for streptomycin, 2.5 for kanamycin, 1.0 for amikacin, 2.0 for ofloxacin, 2.0 for moxifloxacin, 5.0 for ethionamide, and 4.0 for p-aminosalicylic acid. There is no recommended critical concentration for cycloserine [15].

#### Deoxyribonucleic Acid Polymerase Chain Reaction

The *rpoB* gene of *M tuberculosis* isolates were amplified using published primer sets *rpoB*-F 2-(5’-GAG GGT CAG ACC ACG ATG AC-3’ and *rpoB*-R (5’-GAG CCG ATC AGA CCG ATG T-3’) corresponding to nucleotide positions 1030 to 1049 and 1460 to 1478 of the H37Rv numbering system (GenBank). Amplification was performed in a final mastermix reaction volume of 50 µL using a GeneAmp PCR System 9700 thermocycler (Applied Biosystems). Positive and negative controls were used during the polymerase chain reaction amplification process.

#### Deoxyribonucleic Acid Sequencing

Deoxyribonucleic acid sequencing was performed in an automated DNA sequencer ABI 3500 (Applied Biosystems) using the BigDye Terminator v3.1 sequencing kit and primers. Sequenced products (10 µL) were purified in a 96-well plate using BigDye XTerminator purification solution (ThermoFisher Scientific). The 96-well sample plate was loaded onto the ABI 3500 automated DNA sequencer. The DNA sequences were further analyzed and compared with the wild-type sequence of the well characterized *M tuberculosis* H37Rv reference strain using Geneious software (version 10.1.3). Codons with nucleotide changes that differed from the control strain sequence were analyzed for possible amino acid changes conferring resistance. The *Escherichia coli* numbering was used to identify the *rpoB* codon number.

### Clinical Data

All patients showing rifampicin resistance on the MTBDR*plus* assay were started on standard MDR-TB treatment. During the study period, such treatment consisted of kanamycin for the first 6 months plus moxifloxacin, ethionamide, terizidone, ethambutol, and pyrazinamide for 18 to 24 months. Tuberculosis clinical data, including age, gender, HIV status, CD4 count, antiretroviral treatment, previous TB history, and treatment outcomes, were obtained from the MDR TB electronic data system of the provincial Department of Health. Treatment outcomes were defined according to standardized international consensus [16]. Cure and treatment completion were considered successful treatment outcomes, whereas death, treatment failure, and interruption/loss to follow up were classified as unsuccessful treatment outcomes.

### Data Analysis

Continuous variables such as age were summarized using median (interquartile range [IQR]) and compared using Student’s *t* test or Wilcoxon Mann-Whitney test as appropriate. Categorical variables, such as sex, were summarized using percentages and compared using χ^2^ test or Fisher’s exact test, as appropriate. The χ^2^*t* test was used to calculate association between rifampicin MIC and INH resistance. A Student’s *t* test was used to determine whether there was a statistically significant difference between MIC agar dilution (AD) for discordant and susceptible isolates. A one-way analysis of variance was used to determine whether there was a statistically significant difference in mean MICs of isolates with 3 different mutations (Q513P, D516V, and D516Y). Pearson’s correlation was used to calculate linear correlation between MIC AD and MIC MGIT. All analyses were conducted using SPSS version 25 (IBM Corporation). The level of significance was set at *P* < .05.

### Ethics Considerations

Ethical clearance was obtained from the University of KwaZulu-Natal Biomedical Research Ethics Council (BE418/16). Because these were program data, no individual patient consent was required, but permission was obtained from the KZN Department of Health (HRKZ343/16).

## RESULTS

Between May and December 2014, 1302 cases of rifampicin-resistant TB were identified using MTBDR*plus* 2.0. Of these, 60 were susceptible to rifampicin on the APM, a discordance prevalence of 4.6% (95% confidence interval [CI], 3.5%–5.7%). After adding 23 additional discordant isolates from storage, a total of 83 discordant isolates from 83 patients were further analyzed. Of these, 51 (61%; 95% CI, 50.5%–71.5%) were susceptible to INH. In contrast, only 236 (19%; 95% CI, 16.8%–21.2%) concordant rifampicin-resistant isolates during this time were susceptible to INH.

**Figure 1.**
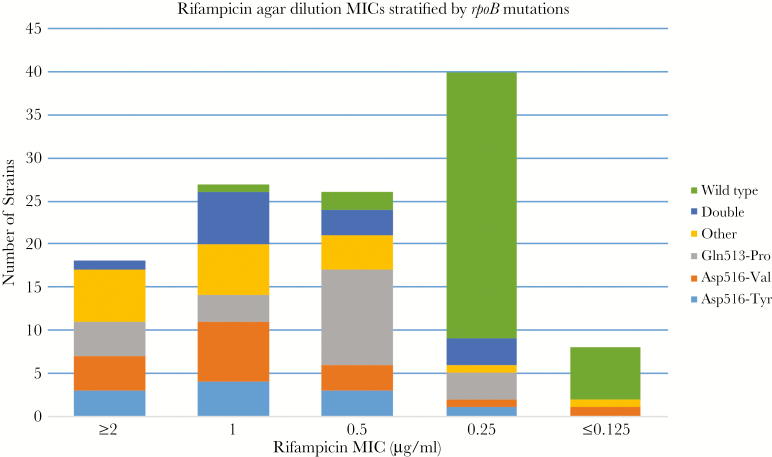
Rifampicin minimum inhibitory concentration (MIC) distribution determined using agar dilution on discordant and susceptible (wild type) isolates stratified by *rpoB* mutation. Although most of the susceptible isolates fall within an MIC of ≤0.25 µg/mL, there is some overlap with the MICs of the discordant isolates.

The isolates displayed a wide range of MICs with the majority centered around the critical concentration of 1 µg/mL (Table 1). The mean MIC for AD, MGIT, and Sensititre was 0.90 (standard deviation [SD] = 0.64), 0.89 (SD = 0.66), and 0.86 µg/mL (SD = 1.10), respectively, and the difference was not statistically significant (*P *= .97). The mean MIC AD for the fully susceptible isolates (0.26 µg/mL) was significantly lower than that of discordant ones (0.93 µg/mL) (*P *= .0001), with the majority (37 of 40) of susceptible isolates showing a rifampicin MIC of ≤0.25 µg/mL. However, there was some overlap between the MICs of discordant and susceptible isolates (Figure 1). The MGIT MIC of the replicates for the H37Rv were ≤0.125, 0.25, 0.25, 0.25, 0.25, 0.25, 0.25, 0.25, and 0.5 µg/mL.

**Table 1.** *rpoB* Mutations of 83 *Mycobacterium tuberculosis* Isolates, Their Rifampicin and Rifabutin MIC Distribution Plus Isoniazid Susceptibility Results

MIC µg/mL					
RIF AD	RIF MGIT	RIF Sensititre	RFB Sensititre	*rpoB* Mutation	INH
0.25	0.25	0.25	≤0.12	His526Ser	S
0.5	1	ND	ND	His526-Arg and His526-Tyr	S
2	0.5	0.5	≤0.12	Gln513-Pro	S
2	2	0.5	≤0.12	Asp516-Val	R
2	2	ND	ND	Ser531-Leu	S
2	0.5	0.5	≤0.12	His526Leu	S
2	0.25	0.25	≤0.12	His526Leu	R
1	1	0.5	≤0.12	Gln513-Pro	S
2	2	1	≤0.12	Gln513-Pro	S
2	1	2	≤0.12	His526Ser	R
2	0.25	0.5	≤0.12	His526Ser, Asp516Tyr	R
2	0.5	0.5	≤0.12	Asp516-Tyr	S
1	2	4	≤0.12	Leu511Pro	S
2	2	2	≤0.12	Asp516-Tyr	S
2	2	4	≤0.12	His526Leu	R
0.5	>2	0.25	≤0.12	Leu511Pro	S
1	2	ND	ND	Asp516-Tyr	S
1	2	2	≤0.12	His526Leu	R
0.5	1	0.25	≤0.12	Asp516-Tyr	R
1	1	ND	ND	Asp516-Tyr	S
1	2	2	≤0.12	Leu511Pro	R
1	0.5	0.5	≤0.12	Asp516-Tyr	R
1	2	1	≤0.12	Asp516-Tyr and Asp516-Val	R
0.5	0.5	1	≤0.12	His526Leu	S
0.5	0.5	0.25	≤0.12	Gln513-Pro	S
0.25	0.5	0.5	≤0.12	Ser509-Thr and Gln513-Pro	S
0.25	0.25	0.5	≤0.12	Asp516-Val	R
0.25	0.25	0.25	≤0.12	Ser509-Thr and Gln513-Pro	S
0.5	0.25	0.25	≤0.12	Asp516-Tyr	S
0.5	0.25	0.5	≤0.12	His526-Arg	R
0.25	0.25	≤0.12	≤0.12	Asp516-Tyr	R
1	2	1	≤0.12	Asp516-Val	R
2	2	1	≤0.12	Asp516-Val	R
0.5	1	8	0.5	His526-Arg and His526-Tyr	S
1	0.5	0.5	≤0.12	Asp516-Val	S
0.25	0.5	0.5	≤0.12	Gln513-Pro	R
0.25	0.25	0.5	≤0.12	Gln513-Pro	S
>4	>2	1	≤0.12	Asp516-Val	R
2	2	2	0.25	Leu511Pro	S
1	1	1	0.25	Leu511Pro	R
0.25	0.25	≤0.12	≤0.12	Ser531-Pro	S
1	0.5	1	≤0.12	Asp516-Tyr	S
1	1	0.5	≤0.12	Leu511Pro	S
0.5	0.5	0.5	≤0.12	Gln513-Pro	S
0.5	0.5	1	≤0.12	Asp516-Tyr	R
0.5	0.5	1	≤0.12	Asp516-Val	S
0.5	0.5	0.5	≤0.12	Gln513-Pro	S
0.5	0.25	≤0.12	≤0.12	Asp516-Val	S
1	1	0.5	≤0.12	Gln513-Pro and Ser531-Pro	R
0.5	0.5	0.5	≤0.12	Gln513-Pro	S
1	2	1	0.5	Asp516-Val and Asp516-Tyr	R
0.5	0.5	0.5	≤0.12	Ser509-Thr	S
1	0.5	1	≤0.12	Ser531-Leu	R
0.5	1	0.5	≤0.12	Leu511Pro	S
2	2	2	0.25	Asp516-Tyr	R
0.5	1	0.5	≤0.12	Gln513-Pro	S
1	ND	0.5	≤0.12	Asp516-Val	S
0.5	0.5	0.5	≤0.12	Asp516-Val	S
1	0.5	0.5	≤0.12	Gln513-Pro	S
0.5	1	0.5	≤0.12	Gln513-Pro	S
1	0.5	0.5	≤0.12	Gln513-Pro	R
1	0.5	0.5	≤0.12	His526-Arg and His526-Tyr	S
1	1	0.5	≤0.12	Asp516-Val	R
1	1	0.5	≤0.12	Asp516-Tyr and Asp516-Val	S
0.5	1	≤0.12	≤0.12	Asp516-Tyr and Asp516-Val	S
0.5	0.5	≤0.12	≤0.12	Gln513-Pro	S
1	2	1	1	Asp516-Val	R
2	0.25	0.5	≤0.12	Gln513-Pro	R
0.25	0.25	0.25	≤0.12	Gln513-Pro	R
2	0.25	1	≤0.12	Gln513-Pro	R
0.5	1	0.25	≤0.12	Gln513-Pro	R
0.5	1	0.5	≤0.12	Gln513-Pro	S
2	2	2	≤0.12	Asp516-Val	S
≤0.125	≤0.125	≤0.12	≤0.12	Asp516-Val	S
1	1	0.5	≤0.12	His526-Arg and His526-Tyr	S
0.25	≤0.125	≤0.12	≤0.12	Ser531-Leu	S
0.25	0.25	≤0.12	≤0.12	Asp516-Tyr and Asp516-Val	S
0.5	0.5	2	0.25	Gln513-Pro	S
1	2	0.5	0.12	Asp516-Val	R
0.5	0.25	0.5	≤0.12	Gln513-Pro	S
1	1	0.5	≤0.12	Asp516-Val	R
0.5	0.5	0.5	0.25	His526-Leu	S
≤0.125	≤0.125	1	1	ND	S

Abbreviations: AD, agar dilution; INH, isoniazid; MGIT, Mycobacteria Growth Indicator Tube; MIC, minimum inhibitory concentration; ND, not done; R, resistant; RFB, rifabutin; RIF, rifampicin; S, susceptible.

**Figure 2.**
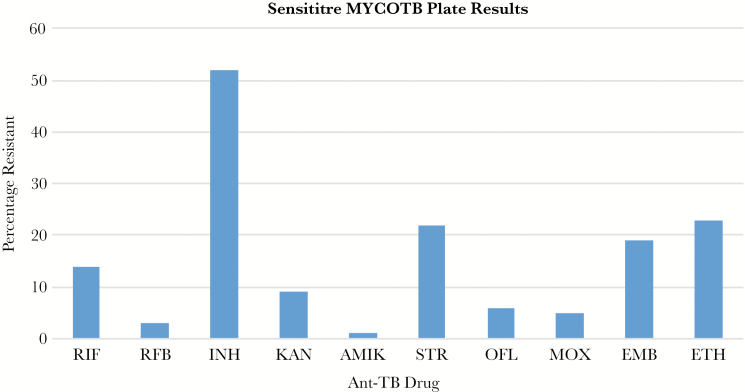
Sensititre MYCOTB plate results showing percentage of *Mycobacterium tuberculosis* resistance against anti-tuberculosis (TB) drugs. AMIK, amikacin; EMB, ethambutol; ETH, ethionamide; INH, isoniazid; KAN, kanamycin; MOX, moxifloxacin; OFL, ofloxacin; RFB, rifabutin; RIF, rifampicin; STR, streptomycin.

Among 79 results obtained on the Sensititre plate (4 were excluded due to contamination), all but 2 were susceptible to rifabutin (97.5%; 95% CI, 94.1%–100.0%) with 69 (87.3%; 95% CI, 79.7%–94.6%) having an MIC of ≤0.12 µg/mL. Figure 2 shows percentage of resistance against anti-TB drugs tested using the Sensititre. The number of isolates that were resistant to the other TB drugs was 41 for INH, 15 for ethambutol, 5 for ofloxacin, 4 for moxifloxacin, 17 for streptomycin, 1 for amikacin, 7 for kanamycin, and 18 for ethionamide. Results for p-aminosalicylic acid were unreliable because there was no agreement between the 2 independent readers.

Sequencing confirmed the presence of *rpoB* mutations in all discordant isolates. The most frequent mutation was the Q513P, which was found in 21 (25.3%; 95% CI, 16.0%–34.7%), followed by D516V in 16 (19.2%; 95% CI, 10.7%–27.7%) and D516Y in 11 (13.3%; 95% CI, 6.0%–20.6%) isolates (Table 1). When looking at the top 3 most frequent mutations, the mean MIC AD was 0.82 µg/mL (SD = 0.62), 0.99 µg/mL (SD = 0.60), and 1.07 µg/mL (SD = 0.65) for Q513P, D516V, and D516Y, respectively, and these did not show any statistically significant difference (*P *= .53). There were also 13 isolates with double mutations. Patients infected with TB strains that had double mutations were more likely to have a previous history of TB (*P *= .009).

### Clinical Characteristics

Clinical characteristics are shown in Table 2. Sixty-one (73%; 95% CI, 61.9%–84.1%) patients were found in the drug-resistant treatment register. The median age was 33 years (IQR = 14) and males constituted 72% (95% CI, 60.7%–83.3%) of patients. Forty-three (71%; 95% CI, 59.6%–82.4%) patients were HIV infected, 37 (86%; 95% CI, 75.6%–96.4%) of whom were on antiretroviral therapy (ART). The median CD4 count was 244 cells/mm^3^ (IQR = 287). A total of 29 (48%; 95% CI, 35.5%–60.5%) patients had a history of previous TB. Thirty-two patients (53%; 95% CI, 40.5%–65.5%) had successful treatment outcomes, whereas 29 (47%; 95% CI, 34.5%–59.5%) had unsuccessful outcomes. These outcomes were comparable to those all rifampicin-resistant TB in 2014, which showed successful outcomes in 59% (95%; CI, 57.0%–61.0%) of patients.

**Table 2.** Clinical Characteristics and Treatment Outcome of Cases with Discordant TB

Characteristic	Number (Total 61)	Percentage	95% CI
Male	44	72	60.7–83.3
Age (median, years)	33 (IQR = 14)		
HIV positive	43	71	59.6–82.4
ART	37	86	75.6–96.4
CD4 count (median, cells/mm^3^)	244 (IQR = 287)		
Previous TB	29	48	35.5–60.5
Positive smear microscopy	33	54	41.5–66.5
Outcome			
Cured	26	43	30.6–55.4
Treatment completed	6	10	2.5–17.5
Defaulted (LTFU)	21	34	22.1–45.9
Died	5	8	1.2–14.5
Failed treatment	3	5	0.5–10.5

Abbreviations: ART, antiretroviral therapy; CI, confidence interval; HIV, human immunodeficiency virus; IQR, interquartile range; LTFU, long-term follow up; TB, tuberculosis.

## DISCUSSION

This study confirms that discordance between molecular and phenotypic methods in the detection of rifampicin susceptibility was indeed caused by mutations present within the *rpoB* region. The prevalence of discordant mutations was 4.6%, which is lower than what has been reported in other countries (ie, ≥10%) [8, 10]. The lower rate found in this study may be due to the fact we used solid agar-based assays for rifampicin susceptibility testing instead of the widely used broth-based methods, which are more likely to demonstrate rifampicin susceptibility in the presence of discordant *rpoB* mutations compared with agar-based methods [6, 7]. Rigouts et al [7] showed that the automated MGIT 960 system was more prone to demonstrate rifampicin-susceptible results in the presence of discordant *rpoB* mutations compared with DST done on Lowenstein-Jensen (LJ) medium. Likewise, in a study involving Supra-National TB Reference Laboratories of the WHO, radiometric BACTEC 460TB and BACTEC 960 MGIT methods demonstrated more rifampicin-susceptible results with discordant *rpoB* mutations compared with the LJ and Middlebrook 7H10 agar [6].

Rifampicin DST is generally believed to be very reliable and reproducible with an average sensitivity for detection of resistance of 97.2% [17]. A study done by Schön et al [18] showed a clear demarcation between the MIC distributions of wild-type strains and those with rifampicin resistance-conferring *rpoB* mutations. Unfortunately, discordant strains do not demonstrate the same distinction. Our study showed MICs bordering the critical concentration of 1 µg/mL and overlapping with those of the wild-type strains (Figure 1). Thus, discordant strains present 2 challenges for phenotypic DST. The first one pertains to the reproducibility of the results, because even a small variation in the MIC (one 2-fold MIC dilution), which is considered acceptable, may change the categorical result between resistant and susceptible. The second problem is caused by overlapping MICs between discordant and wild-type isolates. Several authors have suggested lowering the rifampicin critical concentration to match the epidemiological cutoff (ECOFF) of 0.25–0.5 µg/mL [18, 19]. However, our study shows that even at a tentative breakpoint of 0.25 µg/mL, some discordant isolates will still be missed, whereas some susceptible isolates will be misclassified as resistant. Therefore, routine phenotypic DST is inadequate in the diagnosis of discordant strains.

Similar to rifampicin, the rifabutin critical concentrations of 0.5 µg/mL is higher than the ECCOF of 0.064–0.1 µg/mL [19, 20]. Ängeby et al [20] described TB strains with rifampicin resistance conferring *rpoB* mutations that appeared susceptible to rifabutin, although their MICs were above the ECCOF (0.12–0.25 µg/mL), which suggest that this may be due to a breakpoint artefact rather than true susceptibility. Nevertheless, our discordant strains appear to be truly susceptible to rifabutin, because almost 90% had a rifabutin MIC of ≤0.12 µg/mL—the lowest MIC tested on the Sensititre.

It is well known that certain *rpoB* mutations confer different levels of resistance to rifampicin [21, 22]. The mutation position and the amino acid change determine the level of resistance. Mutations S531L, H526Y, and H526D are associated with high level of resistance, whereas D516V and L511P, D516Y, H526L, H526N, and L533P tend to cause moderate and low-level resistance, respectively [6–10, 21–23]. The Q513P mutation is an uncommon mutation that is largely found in concordant rifampicin-resistant strains [7, 23]. Consequently, its common occurrence among discordant strains in this study raised concerns about the possibility of primary transmission.

Rifampicin resistance has previously been considered a reliable proxy for MDR-TB due to its association with INH resistance in more than 90% of cases [24]. However, the drug-resistant TB survey conducted in South Africa between 2012 and 2014 showed an increase in rifampicin monoresistance (RMR) in KwaZulu-Natal with 39% of all rifampicin-resistant TB being monoresistant. This was particularly pronounced among new cases, so transmission was thought to be the main cause of this increase [25]. Our study showed a 3 times higher rate of RMR among discordant strains than that found among concordant rifampicin-resistant strains. Furthermore, RMR was more commonly found among new TB cases (62.5%; 95% CI, 45.7%–79.3%) with no previous TB, compared with previously treated cases (37.5%; 95% CI, 20.7%–54.3%; *P *= .047), which further supports the contribution of primary transmission in the emergence of discordant TB.

There were 13 patients with TB strains that had double *rpoB* mutations. Jing et al [26] found that double mutations were more common among *rpoB* mutations that confer low-level resistance compared with those with high level resistance. It is understood that initially, a single low-level resistance mutation occurs, but exposure to rifampicin leads to the development of the second mutation. The majority (80%; 95% CI, 55.2%–100%) of our patients with double mutations had a previous history of TB, so accumulation of resistance due to prior exposure to TB treatment may have contributed to their development.

The optimal treatment for rifampicin-discordant TB is unknown, but treatment failure has been reported with rifampicin-based therapy [9–12]. With the recent institution of the new treatment guidelines, these patients are eligible for the standardized shorter MDR-TB regimen [27]. Nevertheless, even this treatment regimen remains longer and more toxic compared with the standard first-line therapy. Previous studies have proposed that in view of rifampicin pharmacokinetic/pharmacodynamic data, a high-dose rifampicin (900 or 1200 mg) may be used to attain the concentration-time curve over the MIC (area under the curve/MIC) of 271 that is required to overcome the discordant TB strains [10, 11, 28]. This remains to be proven in clinical studies.

A large proportion of the discordant strains in our study is still susceptible to first-line anti-TB drugs with 61% showing susceptibility to INH. In a study done by Shah et al [29], 2 patients with discordant strains that were susceptible to INH showed successful treatment outcomes with the standard 6 months first-line regimen, whereas all (5) those who had unsuccessful treatment outcomes had INH resistance. Likewise, Ocheretina et al [9] reported 2 patients with discordant mutations that were susceptible to INH who were cured using the standard first-line therapy. Therefore, it appears that in the presence of INH susceptibility, first-line therapy may still be an option. Given the high susceptibility to rifabutin found in this study, as well as the high HIV/TB coinfection in this region, rifabutin-based first-line therapy may be a better option because it presents fewer interactions with ART than rifampicin [30].

There are several limitations in this study. As previously mentioned, agar-based phenotypic DST was used to screen for discordance. Therefore, this may have selected out other mutations that are only susceptible on the liquid-based assays. In addition, the use of retrospective clinical data means that its accuracy is dependent on the available information. Nevertheless, this study provides an extensive description and characterization of rifampicin- discordant strains in an area with a high burden of drug-resistant TB and HIV.

## CONCLUSIONS

Although the introduction of rapid molecular tests has brought much needed improvement in the diagnosis of drug-resistant TB, discordance between genotypic and phenotypic tests poses new challenges in the interpretation of these results. Our study shows that routine phenotypic and genotypic assays are inadequate in solving this dilemma. The WHO’s recently released guide on interpretation and reporting of LPAs recommends sequencing of the *rpoB* gene where resistance is inferred by absence of binding to the wild-type probe without binding to the specific mutation [31]. Although sequencing may be ideal, with an additional advantage of being able to detect *rpoB* mutations outside the RRDR that are currently missed by the routine molecular tests [32], it is still not readily available in routine clinical microbiology laboratories of high TB-endemic areas where it is needed the most. Finally, there is a need to explore the potential utility for rifabutin-based first-line therapy in patients with rifampicin-discordant TB.
